# MULTIDIMENSIONAL EVALUATION OF BIOLOGICAL AGE USING NMR METABOLOMICS AND ANTHROPOMETRY IN 35,541 ADULTS IN MEXICO

**DOI:** 10.1093/geroni/igad104.1738

**Published:** 2023-12-21

**Authors:** Omar Yaxmehen Bello-Chavolla, Carlos Fermin-Martinez, Luisa Fernández-Chirino, Daniel Ramírez-García, Neftali Antonio-Villa, Alejandra Núñez-Luna, Paulina Sánchez-Castro, Martin Roberto Basile-Alvarez

**Affiliations:** Instituto Nacional de Geriatría, Mexico City, Distrito Federal, Mexico; School of Medicine, National Autonomous University of México (UNAM), Mexico City, Distrito Federal, Mexico; Instituto Nacional de Geriatría, Mexico City, Distrito Federal, Mexico; Universidad Nacional Autónoma de México, Mexico City, Distrito Federal, Mexico; Instituto Nacional de Cardiologia Ignacio Chavez, Mexico City, Distrito Federal, Mexico; Instituto Nacional de Geriatría, Mexico City, Distrito Federal, Mexico; Instituto Nacional de Geriatría, Mexico City, Distrito Federal, Mexico; Instituto Nacional de Geriatría, Mexico City, Distrito Federal, Mexico

## Abstract

Aging occurs across different domains at the individual level, encompassing distinct physiological systems which can be captured using biological age (BA) metrics. Second generation BA metrics are aimed at proxying BA using 10-year mortality risk and include PhenoAge and AnthropoAge. Here, we explored the complementary application of AnthropoAge and an NMR-Metabolomics derived BA measure to explore aging in body composition and metabolic domains in a subsample of 35,541 participants of the Mexico City Prospective Study. Using LASSO Cox regression, we identified 17 metabolites associated with all-cause mortality, in men and 75 in women. We then fitted age-adjusted Gompertz regression models and identified 7 unique metabolites for men and 15 for women. We transformed the results from these regressions to define MetaboAge. Both AnthropoAge (AUC 0.784, 95%CI 0.778-0.790) and MetaboAge (AUC 0.808, 95%CI 0.802-0.814) showed superior performance for prediction of all-cause mortality compared to chronological age (AUC 0.779, 95%CI 0.773-0.786). By regressing AnthropoAge and MetaboAge onto chronological age we obtained acceleration metrics (≥0 defined accelerated aging). By combining acceleration metrics from anthropometry and NMR-metabolomics we identified distinct trajectories of all-cause mortality. Notably, cases with acceleration only in AnthropoAge (HR 1.43, 95%CI 1.33-1.54) and those with acceleration only in MetaboAge (HR 1.86, 95%CI 1.73-2.01) had higher risk of all-cause mortality compared to non-accelerated cases but lower compared to cases with acceleration in both metrics (HR 2.28, 95%CI 2.12-2.45). Multidimensional evaluation of BA may prove useful to capture heterogeneity of aging in diverse populations by capturing aging at distinct physiological domains.

